# Who Cares How Information Feels? A Call for Digital Influence Literacy

**DOI:** 10.1007/s41649-024-00350-0

**Published:** 2025-06-06

**Authors:** Theresa M. Senft

**Affiliations:** Department of Media, Communications, Creative Arts, Language, & Literature (MCCALL), Macquarie University, Sydney, Australia

**Keywords:** Digital influence literacy, Digital platforms, Media literacy

## Abstract

This article introduces digital influence literacy, arguing for its inclusion in programs devoted to lessening the spread of health misinformation online. Influence literacy can be roughly understood as the capacity to recognise, analyse, navigate, and emotionally regulate feeling as it is generated, circulated and monetized over digital platforms, alternately experienced by social media users as mood, movement, sentiment, or environmental vibe. Combining insights from communications, social and behavioural psychology, digital design, and trauma studies, influence literacy can be used to better understand events like #FilmYourHospital, where a single rumour on Twitter wound up feeding into a global conspiracy. It can also be used to better appreciate how trends, memes, challenges, and calls for justice move from online spaces to offline ones. After arguing that traditional media literacy’s assumptions about the value of emotional communication require substantial re-thinking in the age of platforms, this article lays the groundwork for topics that might be included in discussions on influence, moving from psycho-social theories of feeling to techno-social operations like emotion recognition, sentiment mining, persuasive computing and emotion optimization on platforms. To assist those looking to add influence literacy to classrooms, a teaching framework called the Influence Ecosphere is offered, with discussion topics suggested to help supplement media literacy’s traditional focus on rights with a feeling-based ethics of care.

## Introduction

This perspective article introduces *digital influence literacy*, urging its inclusion in media literacy efforts, especially those tasked with stemming health misinformation online. Influence is the capacity to affect someone or something. While teaching at a university in Australia and advising public health agencies about social media, I have seen people frequently equate digital influence with charismatic individuals, as in “influencers”. I understand it as a dynamic between individuals, groups, institutions, and technological systems, moving from online to offline environments, and back again.

This special issue concerns the ethics of governing artificial intelligence in healthcare, where recommendations are increasingly influenced by predictive algorithms sifting large data sets. Too often, these overlook outliers—vulnerable members of society—or anyone at vulnerable moments (Villegas-Galaviz and Martin 2023). Consider the World Health Organization’s argument that the decision by social platforms to algorithmically push content with high watch time influenced an “infodemic” among people seeking health guidance online (2023a).

In efforts to fight the infodemic, it is common to hear calls for more digital media literacy. To date, these have largely ignored influence literacy: the capacity to recognise, analyse, navigate, and emotionally regulate feeling as it is generated, circulated and monetized over digital platforms as mood, movement, sentiment, or vibe. This gap has been noted by those urging that media literacy take more seriously the intertwining of spectacle and mistrust in digital landscapes (Mihailidis 2019); focus more attention on “feelings generated by news, not just the facts provided within it” (Sivek 2018, 124); and offer students tools for managing emotions online (Lincenberg 2021) beyond advice to better curate, digitally detox, or in the words of Gen Z, “touch grass”.

This article contains four sections. The first examines traditional media literacy assumptions about the nature and value of emotional expression in public life, arguing these require substantial rethinking in the age of social platforms. The second considers how emotion shapes the way humans receive and respond to information, briefly overviewing theories of appraisal, priming, affect heuristics, normativity, and social contagion. The third details how digital platforms influence users through emotion recognition, sentiment analysis, and engagement optimization. To assist with teaching influence literacy, the final section introduces the Influence Ecosphere, a framework combining insights from communications, social and behavioural psychology, persuasive computing, and trauma studies.

As a neurodiverse survivor of childhood violence, I hope these perspectives resonate with many, but especially the 70% who have experienced trauma (Kessler et al. 2017), the 15% navigating neurodevelopmental disorders like autism and ADHD (World Health Organization 2023d), and/or the one in seven young people suffering with mental health conditions like anxiety and depression worldwide (World Health Organization 2023c, 2024).

## Emotion: Media Literacy’s Discontent

Although media literacy has no globally accepted definition (Yue 2022), it is commonly understood as the ability to access, analyse, and produce information (Aufderheide 1993). Digital media literacy content can include explanations of algorithmic tracking and data protection on platforms. Competencies vary from learning to fact-check images through reverse-searching, to training in “netiquette” norms.

Like most theories, media literacy is largely “a value-added export from Euro-America” (Jackson 2019, 63) with regional variations in practice. North American models stress “critical autonomy in relationship to all media” (Aufderheide 1993, 1), demanding informed consumption and freedom to issue counter-messaging, usually as a critique (Jeong et al. 2012). In the Asia Pacific region, programs stress media literacy’s importance to the “participation, connection, and well-being” of citizens (Yue 2022, 191).

Wherever taught, the civic wager of literacy programs is that once people understand systems, skills and norms, they will interact with greater rationality. To settle arguments online, students learn debunking, which involves evaluating evidence against the authority of sources trusted by all. During the pandemic, digital media literacy experts advocated “pre-bunking” games as psychological inoculation (Roozenbeek et al. 2020)—an epidemiological spin on the hope that with enough communicative rationality, the internet can function like a true public sphere (Schaefer et al. 2013).

Online and off, there is value in teaching people how to check facts, evaluate arguments, and deliberate politely. Of rapidly diminishing value, however, is teaching that ignores the realities of social media platforms, where misinformation is frequently circulated by users who insist they *are* making rational arguments, endorsed by authorities *they* trust (Boyd 2017). In a recent study of online political discussions across Asia, researchers offer two possible reasons for this dynamic, which they call *misinformed choice*: the power of self-reflective information loops and the ferocity with which users condemn others as fake news spreaders (Berger and Rother 2023; Majumdar 2024a, b).

Far from being exclusive to Asia, the blaming and shaming of misinformed choices increasingly typifies public online discussions of Western academic research. A recent article for *The Conversation* discussed a viral challenge called #FilmYourHospital, where users shared videos of empty hospitals at the pandemic’s height. Originally thinking online bots were responsible for its spread, researchers later learned #FilmYourHospital’s main drivers were influencers with interests in conspiracy theories, using evidence not faked, but misunderstood. With protocols like visitor bans in place, a packed hospital *can* feature empty hallways and parking lots. Doubling down, online commenters insisted, “Calling people conspiracy theorists…seems nothing more than an attempt to discredit those with whom the authors disagree” (Gruzd and Mai 2020).

Here, we see digital media literacy’s first shortcoming: by valorising the power of rationality above all, it leaves people largely unprepared to manage moments where influence operates over social platforms by *affecting affect*—eliciting, spreading, and modulating “social emotions” primarily related to fear, trust, shame, and esteem.

I am not suggesting media literacy scholarship avoids emotion, a concern as old as Aristotle’s *Rhetoric*. Rather than scarcity of theoretical focus, the challenge is one of pedagogical locus. In early education, children are taught that feelings begin in the body and that their emotional reactions impact others (Gilliam 2021; Becerra and Campitelli 2013). To successfully adapt, they learn to emotionally regulate within mediated and other environments (Cracco et al. 2017). Indeed, a child’s ongoing inability to self-regulate is considered a hallmark symptom of possible neurodevelopmental disorders and/or mental health conditions.

After childhood, educational conversations about emotion move from engagement with specific bodies to concerns for abstracted publics. In classes on news, propaganda, and/or the attention economy online, students learn to spot trolling, clickbait and rage farming, mimicking the cool detachment of their teachers as they worry out loud about the emotional influence of these on unnamed others. In Big Critique (Burgess 2023) lectures on algorithmic control, anxiety raised by warnings that nobody escapes techno-determinist abstractions like data capitalism is soothed by workshops on keeping one’s online networks as free as possible from unwanted surveillance, bots and sources. Left unquestioned, hopes regarding the protective powers of detached critique can ossify to convictions about the unreliability of anything beyond one’s personally arranged online networks, as in a recent study showing people are more willing to believe misinformation shared by online strangers to whom they are connected than offline friends (Osatuyi and Dennis 2024).

This mindset has been linked to some troubling political effects worldwide, especially among young people. In the U.K. and Australia, educated young people made up the largest cohort of COVID-19 deniers during the pandemic, in part due to their information-sharing patterns online (Duffy and Allington 2020; Pickles et al. 2020) In Asia, these patterns have been likewise linked to young people’s growing turn toward political conservatism (Laksana 2020), illiberal populism (Said and Berghaus 2024), and “anti-woke” rhetoric (Keegan 2023.) Around the world, young people are at record-high levels of risk for recruitment by extremist groups online, who frequently begin by extending emotional validation for scepticism of all stripes, saving the highest esteem for those willing to reject all authority outside the group. (Lederer 2021; Adams and Sally 2021; UNESCO 2017).

If traditional media literacy spends too much time advocating critical disengagement from emotion, in classes focused on advertising, public relations, and strategic communication, the opposite problem exists. There, appeals to emotion are touted as the premier mechanism for capturing the attention of consumers, clients, patients, and/or stakeholders. On platforms, we are encouraged to view *ourselves* as a target market for newsfeeds and “for you” pages designed to provide us content similar to what we have clicked, dwelled, shared, and commented on in the past, and less of what we have skipped. In a recent article on social media literacy competencies, Schreurs and Vandenbosch (2021, 323) rightly argue that the global reach of platforms means “being literate cannot be about possessing the ‘right’ affective responses but instead relates to managing one’s affective responses”. To this end, they suggest social media literacy competency ought to include increased awareness of the emotional effects of one’s feed, and the skills to curate with an eye toward predominately positive interactions. It is benign enough advice, but for the inference that with a bit of training not unlike cognitive behavioural therapy, people should be able to cultivate their emotional landscapes online as simply as they do off. In actual practice, cognitive behavioural therapy requires substantial modifications to serve all sorts of individuals, especially trauma survivors (Arellano et al. 2014).

This brings us to media literacy’s second problem: its structuring assumption that by and large, people live their lives in emotional neutrality offline, and would do so online, were it not for the informational pollution and incivility permitted on platforms.

Decades ago, proponents of the now discredited hypodermic needle model argued that mass media influences by infusing feelings into otherwise peaceful minds (Tones 1996). Digital literacy programs largely continue to frame feeling as a sort of toxin to be removed, rather than the substance residing at the core of cognition and communication. Consider how phrases like “information overload” miss the vulnerability that comes with having one’s resources overstretched, insinuating that if messages were properly managed, people would receive them in a state of equanimity (Senft and Greenfield 2023).

It is perhaps ironic given a coinage-like infodemic that discussions of trauma are still largely absent from digital literacy programs. Trauma is commonly understood as an ongoing stress response to disaster, violence, abuse, neglect, or other harms. A defining feature of post-traumatic stress disorder (PTSD) is the feeling of being triggered by a memory-related image, sound, or scent, ushering in emotions like panic, anger, and/or overwhelm. (Wright 2021) In environments where PTSD is endemic, feelings like fear, shame, self-blame, alienation, and betrayal dominate (DePrince et al. 2011). Some characterize the pandemic as mass trauma, noting how “people today seem to be gradually moving into a hyper-vigilant stance…” (Horesh and Brown 2020, 322).

Of course, one need not be traumatized to turn to platforms hoping to relieve stress, only to become *more* emotionally distraught. Here, we see digital media literacy’s third shortcoming: its failure to equip users to communicate on platforms fuelled by the algorithmic generation, circulation, amplification and monetization of “emotions on the move” (Boler 2018). This process is detailed later, with four axioms worth underscoring now:

1. Social media’s advertised ethos is one of democratic social connection, but the guiding principle of platforms is algorithmic connectivity: the collection, packaging, and sale of user data for profit.
2. Hoping to keep users generating data for as long as possible, social media platforms frequently deploy algorithms tasked with recognizing content most likely to elicit reactions like outrage, disgust and sometimes laughter among users, amplifying its reach through mechanisms like recommendation pages and news feeds.
3. Behind every user who has gained influence online by “gaming” social media algorithms to spread a message, attract followers, or make money, there is a user who has “been gamed” (made anxious, shamed, harassed, threatened) by the spread of emotion online.
4. Just as positive engagement online can lead to positive experiences offline, negative engagement online can lead to suffering, threats and even physical harm offline.

To summarize, in its current iteration, digital media literacy fails to address the dynamics of social platforms in three respects. First, its continued advocacy of rational argument as the best defence against unwanted influence leaves people largely unprepared to manage influence stemming from the activation of “social emotions” like fear, trust, shame, and esteem. Second, its continued assumption that users begin their time online in a state of emotional neutrality is at odds with how frequently people scroll platforms hoping to alleviate offline stress, and ignores people negotiating conditions like trauma, depression, anxiety, and/or neurodivergence-related challenges, for whom baselines of emotional reactivity are common. Finally, digital media literacy’s growing penchant for Big Critique can leave students convinced the effects of algorithmic influence lie entirely beyond human control, leading them to side-step consideration of how their arousal might be contributing to a range of troubling behaviours that start online and move off.

Rather than continuing to encourage what increasingly seem like fantasies of rational autonomy within platformed environments dominated by emotional immersion, virality, and misinformed choice, we might instead consider what digital media literacy stressing *critical embeddedness* could look like. This could start with the question of how people come to assume the identity of a “user” on social platforms through a series of interactions designed to invoke “the self and its motivations, choices, and networks” (Cho et al. 2024, 909.) But first, we need to understand emotion.

## From Emotion to Contagion

Emotion, mood, sentiment, and affect are frequently interchanged, but there are distinctions. Emotion tends to be acute and is generally considered a combination of physiological reaction, subjective experience, and frequently, interpersonal expression (Moors 2013). Compared to emotion, moods are more diffuse, sentiment is referenced by valence, and affect includes all of these (Mohammad et al. 2017).

Biologists teach that emotion originates when the brain’s reward pathways are activated. When dopamine is released, pleasure ensues; when oxytocin rises, so do impulses like trust and bonding (Whissel 2023). During short moments of stress, adrenaline floods the nervous system; when danger is perceived as persistent, cortisol keeps the body “revved up and on high alert” (Harvard Health Publishing 2024).

The drive for safety shapes much of how we process information and make decisions. Appraisal theory holds that two people can have different emotional responses after being exposed to the same stimulus, depending on how they feel it relates to safety or goal achievement (Moors et al. 2013) When exposed to new environments, our brain primes itself with emotional memories of the past to determine how we should behave in the present (Moors et al. 2013) Priming can have predictive force: when we associate someone with positive emotion, we instinctively trust them more, and vice versa (Clark and Taraban 1991). According to affect heuristic theory, we shortcut to these “gut feelings” during decision-making (Slovic et al. 2007).

Emotions feel personal, but as every child learns, communicating emotion is a social affair. Social psychologists use the term *social influence* to describe how we adjust attitudes, thoughts, or behaviours, based on the actions, reactions, or even the mere presence of others (Gilovich et al. 2011). This often involves the operation of social emotions like jealousy, trust, loyalty, and confidence, involving “other people’s thoughts, feelings, or actions as experienced, recalled, anticipated, or imagined” (Hareli and Parkinson 2008, 131).

Social influence is frequently divided into normative and informational variants. Normative influence “stems from our concern about how we appear to others,” while informational influence reflects that we also “have a desire to learn the truth” (Harkins and Williams 2014, 5). Assuming no coercion, people are free to resist or conform to social influence. Conformance is frequently divided into internalization, compliance, and identification (Kelman 1958). A person who internalizes something publicly accepts and privately agrees. A person who complies publicly agrees but does not privately accept. A person who identifies provisionally agrees if it aligns with their understanding of their role within the group.

Researchers have found that offline and online, people mimic or “catch” the emotions of others (Berger 2016). An infamous Facebook study showed when negative sentiment in News Feeds was reduced, users produced more positive content, and vice versa (Kramer et al. 2014). The decision by social platforms to algorithmically amplify messages inciting anger, surprise, disgust or laughter has been justified by research indicating users tend to share this content the most, even when discussing “neutral” topics like science (Milkman and Berger 2014; Scheufele and Krause 2019; Vosoughi et al. 2018.)

Content shared by many over a short period is referred to as viral (Nahon and Hemsley 2013). Emotion contagion features heavily in viral challenges, where users post images or videos of themselves and encourage others to do the same (Abraham et al. 2022) and *viral justice*, in which witnesses share footage of unacceptable behaviour to punish perpetrators, if not in an actual court of law, then in online courts of public opinion, and sometimes places of work, education, and residence (Wood et al. 2019).

In healthcare contexts, viral justice has taken a troubling turn, with users increasingly targeting those with whom they disagree regarding medical testing, masking, and vaccinations. A list of recent harms includes “dehumanizing language on social media posts, threats of physical or sexual violence, coordinated negative reviews of clinicians or medical practices on Yelp and Google, fabricated or misattributed Tweets, and doxing or the public posting of personal information, such as home addresses” (Lalani et al. 2023, 1115). Globally, violence against healthcare workers is at an all-time high, with social media frequently mentioned as the source of exacerbation (Carpiano et al. 2023; Kuhlmann et al. 2023; Eala et al. 2022; Devi 2020).

## The Emotional Internet

To this point, we have briefly discussed how emotion drives people’s relationship to information and one another. We turn now to consider the three most popular technologies through which feeling is tracked, analysed, and marketed on platforms: automated emotion recognition (AER), sentiment analysis, and persuasive computing.

AER involves tracking users’ online expression in a range of formats (e.g.. words written, images produced, face, voice, and/or heart scans) and categorizing these by emotion, generally limited to the so-called big seven: anger, disgust, fear, happiness, sadness, surprise, and neutrality (Boesch 2023). Depending on the source, AER involves machine learning, natural language processing, computer vision, and/or deep learning, and can include operations as varied as inferring expression from a face, the intensity from a voice, and/or the emotional effects of language on audiences—each of which require different methods and ethical considerations (Mohammad 2022).

Sentiment or slant analysis involves assessing whether people associate a product, service, or program with emotional positivity, negativity, or neutrality, in hopes of making accurate predictions about future behaviour (Mohammad et al. 2017). A longstanding practice in commercial fields like marketing, sentiment analysis features now heavily in the public health communications practice known as social listening (World Health Organization 2023b). On social platforms, it is common to see people sharing health-related content that simultaneously criticizes governments, spreads rumours, and asks legitimate questions about medical treatment. Are these properly classified as positive, negative, neutral or something else?

While public awareness of technologies like AER and sentiment analysis certainly exists, it is often eclipsed by concerns about the impact of persuasive computing “intentionally designed to change a person’s attitudes or behaviour in a predetermined way” (Fogg 1998, 225). Common persuasive techniques compiled by Gurgun et al. (2023) include reduction (turning complex decisions into one-click operations), suggestion (offering pre-created default responses), self-monitoring (often through visuals), and recognition (sometimes with marks of esteem like “blue ticks”). Stöcker (2020, 135) compares these to psychologist BF Skinner’s boxes: “Instead of lights, speakers, levers, food pellets and electric shocks, the user interface designers of today employ multimedia content, like, share and reaction buttons, likes, reactions and comments as stimuli, actions and rewards”.

On social platforms, decisions to deploy emotion-centric technologies turn on a financial model known as optimization, which argues that emotionally aroused users stay on platforms longer, generating more data to be sold (Bakir and McStay 2022). Although early research on emotion and dwell time focused on user brain scans showing increases in the “happiness hormone” dopamine (Haynes 2018), today’s models generally favour amplifying negative emotions over positive or neutral ones (Merrill and Oremus 2021). Public fascination with emotion optimization (frequently shortened to “engagement”) has given rise to what Bucher (2018) calls the *algorithmic imaginary*, in which users narrate online behaviour (their own, or others) in terms of what they perceive as algorithmically favoured. For instance, one does not need to identify as an influencer to hold the conflicting beliefs that (1) the quickest way to garner attention online is to post incendiary content, and (2) given that everyone is searchable these days, we should feature our best selves online, expecting others do the same (Senft 2013) .

Ethically speaking, the deployment of technologies of emotion on platforms is arguably neither good nor bad, unless user manipulation comes into play (Botes 2023). Perhaps the most well-known example here involves Cambridge Analytica’s use of Facebook data to politically micro-target voters during the 2016 U.S. elections. Interestingly, newer research shows microtargeting does not necessarily link to success in the polls (Dizikes 2023). Another common concern is that platform recommendation algorithms create what Parisier (2011) calls filter bubbles, where polarizing echo chamber dynamics can predominate (Sunstein 2018). Again, a causal relationship between automated filtering and ideological polarization has yet to be proven, at least in political contexts (Bruns 2019).

It is increasingly difficult to distinguish between technologies that manipulate unaware users, and the refusal of users to address what they know are their habituated relationships to technology. Sometimes these are rationalized through narratives of emotional safety, such as the increased use of features like “hide post” and “unfriend” by users wishing to avoid certain types of content, but uncomfortable engaging in direct confrontation online (Gurgun et al. 2023, 74). Rather than mice trapped in conditioning boxes they cannot escape, a more accurate metaphor for platform users might be swimmers dumped into at times interconnecting pools of consumer taste, sharing patterns, and preferred formats of expression, leading them into environmental conditions they can sometimes predict, and other times not. When users choose or unconsciously default to only recommendations offered, they contribute to pool stagnation, which can bring worrying consequences. One example was the GamerGate controversy, in which young people who believed themselves gathered by a love of gaming found themselves unexpectedly involved in a political grooming operation led by right-wing U.S. extremists (Mortensen 2018).

## Mapping the Influence Ecosphere

Having discussed the psychosocial, technological, and economic dimensions of emotion on platforms, I want to offer a very brief sketch of what teaching influence literacy might include in classroom settings, understanding that classrooms are not enough (Schreurs and Vandenbosch 2021). Here, I focus on content that might be adapted into existing digital media literacy programs. A future article discusses competencies. These include developing the capacity to gauge the impact of emotional flows online; the skills needed to recognise and regulate our own emotional reactivity; the practice of non-violent communication, including the validation of metaphors and/or personal experiences without necessarily capitulating to positions espoused; and introduction to principles of and communities advocating human-centred digital design.

**Fig. 1:**
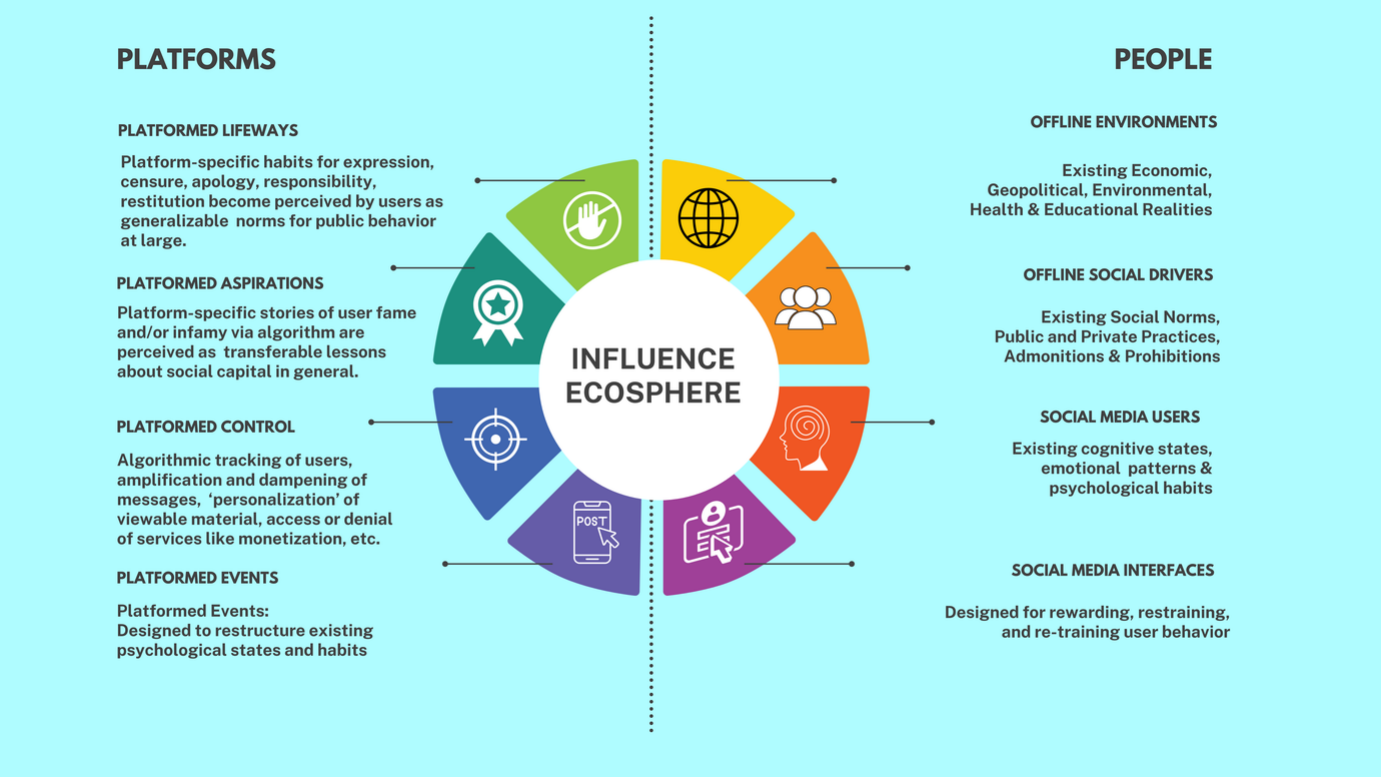
The influence ecosphere (image courtesy of the author)

I share below a framework for teaching influence literacy I call the Influence Ecosphere (Fig. 1). It imagines the “land” of online influence as technologies of emotion, its “water” as economies of optimization, and its “atmosphere” as the sociality of users in locales around the world as they navigate information, behaviour and consequence on and off platforms. In each section, I suggest lecture possibilities and preliminary exercises designed to encourage students to consider their everyday experiences “grappling with, anxious about, or simply uninterested in the possibilities, risks, and challenges of data and automation” (Burgess 2023, 1244). My discussion moves around the diagram clockwise, from people to platforms, with the understanding that influence itself moves in patterns that cannot always be accurately predicted.

The first section, “Offline Environments”, is meant to introduce the paradoxical nature of emotion, which can feel so deeply personal, yet is largely structured by forces outside us. To ground lectures on topics like emotional appraisal, priming, and affect heuristics, instructors might share one personal preference that changed with age and one that has not shifted since childhood, inviting students to share theirs. Follow-up conversations might move from preferences to beliefs, and then values. To encourage reflection on how online environments shape the way they think about themselves and others, students might be asked to individually conduct two Google searches: one for memes relating to the platforms, communities, or online activities they most enjoy and another for memes related to a platform they do not know well or an online activity they do not enjoy. Coming together as a group, the class might spend time evaluating these for perceived accuracies and inaccuracies of stereotypes presented.

The second section, “Offline Social Drivers”, can be used to foreground discussions of social influence, contagion and virality. Here, students might be asked to reflect on the last time they joined (or resisted) a popular challenge online or discuss a time they found themselves provisionally agreeing with a community on a topic, only to change their minds later. A more advanced assignment might require students to develop a class list of institutions and individuals regarded by their families or communities as trustworthy (e.g., media outlets, elected officials, religious leaders, their teachers, physicians, and coaches) reflecting on whether they personally agree with these assessments, and why.

The third section, “Social Media Users”, is where teachers can underscore how issues like emotional health and neurodiversity are central to the study of social media dynamics. A trauma-informed approach might involve asking students to develop a map of words they associate (or have heard associated) with the word “trigger”, relating this to more socially neutral words like “cringe” or “ick”, or phrases like “not passing the vibe check”. After sharing statistics on the global rise of depression, anxiety and neurodevelopmental conditions among young people, students who are willing might be asked to share a time it became clear to them that they were in emotional distress online, how others responded (if they did), and what felt helpful (or not) in that moment.

The fourth and fifth sections move to more technological considerations. “Social Media Interfaces” provide space for lectures on technologies like emotion recognition, sentiment analysis, persuasive computing and emotion optimization. “Platformed Events” provides a moment for students to explore what they have learned, perhaps by charting in real-time their physical and emotional responses to notifications, announcements, or AI-generated messaging online.

The sixth and seventh sections explicitly consider the role of algorithms in generating and distributing emotion online. “Platformed Control” can be where students consider emotion optimization as a private financial model with increasingly public implications. Examples worth discussing include the pressure placed on platforms during the pandemic to tag COVID-19-related misinformation, or a recent law banning Australians under 16 from (selected) platforms, framed as an anti-bullying initiative. (Butler 2024).

“Platformed Aspirations” can be a good place to discuss algorithmic imaginaries. Here, students might be asked to analyse a post gone viral, drawing on viewer metrics and comments (especially ones like “this popped up on my feed”) to observe which perspectives seem to dominate, and which seem muted or entirely absent. From here, a conversation might be had about which techniques for attracting notoriety or avoiding censure have moved from online spaces to offline ones, as in phrases like “Pics or it didn’t happen” or “Don’t cancel me for this, but…”

Section eight, “Platformed Lifeways” allows for discussions of the consequences of expression and behaviour that increasingly traverse from online to offline spaces, and back again. In healthcare contexts, a particularly interesting example of viral justice worth discussing is the case of teen vaccine advocate Ethan Lindeberger, who rose to internet fame through his questions on Reddit, only to become the target of anti-vax doxing and death threats (Thielking 2019).

## Conclusion

In 2024, anyone can discover they have inadvertently circulated misinformation online, not because we are uninterested in facts, but because platforms frequently leave us too emotionally overstimulated to do otherwise. For digital media literacy to be of value to those seeking information and interaction on platforms, it must relinquish its inherited belief that people share the same flows of content, networks of trust, and baselines of emotional neutrality; that debunking and debate remain the gold standard for communicating about information in public settings; and that students are better served by a pedagogy that promotes critical autonomy as the best way to protect ourselves from the techno-social spread of emotion online, than one that starts by admitting its psychological, physiological, social, and political effects on us all.

This article advocates digital influence literacy to help users recognise, analyse, navigate, and manage the emotional regulation of feeling as it is generated, circulated and monetized over social media platforms. To assist teachers with discussions of psycho-social theories of emotion and platform operations like emotion recognition, sentiment mining, persuasive computing, and engagement optimization, I offered a preliminary teaching framework called the Influence Ecosphere, with more developed curricular offerings to follow.

Traditionally, digital media literacy pedagogy has emphasized people’s right to online access, skills, and understanding of environments. While still vitally important, in matters relating to digital influence, “an ethic of care is arguably more important than a rights-based approach, or at least, crucial to its achievement” (Jakimow et al. 2019, 281). For teachers, frequently torn between professional duties of care, legal obligation to care, and the fact that they must at some point care for themselves, this can be easier said than done (Pepper 2021).

As an educator, it has been humbling to realize that each time I advocated emotional detachment as the best hope for navigating information, audiences, and technologies on platforms, I may have inadvertently harmed emotionally vulnerable students, possibly leading them to harm others beyond my classroom. Not unlike algorithmic decision-making of which we are right to be wary, I have used justifications of time and resource limits to sideline the needs of those I have been told are data outliers, ironically enough including myself. I could have chosen otherwise yesterday. I try to choose differently today.

## Data Availability

No new data were created or analysed during this study. Data sharing is not applicable to this article.
